# Supplementary material for: A stakeholder group assessment of interactions between child health and the sustainable development goals in Cambodia
Source: Commun Med (Lond). 2022 Jun 16;2:68. doi: 10.1038/s43856-022-00135-2 (PMC9203784; doi:10.1038/s43856-022-00135-2)
Supplement: Supplementary file 2 — Supplementary information [file 43856_2022_135_MOESM2_ESM.pdf]

Supplementary Information

Table of Contents

Supplementary Note 1. Key indicators and the SDGs in Cambodia 2
Supplementary Figure 1. Child mortality trends over time in Cambodia. 2
Supplementary Figure 2. Correlation between the Cambodian Sustainable Development Goals (CSDGs). 4
Supplementary Figure 3. Policy overview and developmental context of Cambodia. 5
Supplementary Note 2. Budget analysis 6
Supplementary Figure 4. Annual expenses in 2010 USD between 2000-2013. 6
Supplementary Figure 5. Procent of total annual expenses in 2010 USD between 2000-2013 for selected ministries. 7
Supplementary Methods. Overivew of participants and details on the cross impact matrix and network analysis following the SDG Synergies approach. 8
Supplementary Table 1. List of stakeholders that participated in the study. 8
Supplementary Figure 6. Illustration of the difference between first order and second-order influence, and how second-order influence is calculated. 10
Supplementary Figure 7. Illustration of second-order interaction and aggregated second-order influence from goal A on goal D 11

Supplementary Note 1. Key indicators and the SDGs in Cambodia

There are many data gaps regarding the Sustainable Development Goals (SDGs) and the Cambodian Sustainable Development Goals (CSDGs). To illustrate what does exist however, we present child mortality trends in Supplementary Figure 1A and 1B, and a Pearson paired-observational correlation analysis in Supplementary Figure 2 based on key indicators for the CSDGs which can be found in Supplemenary Data 1. In addition to this, we have collected all available historic data from the SDG Indicator database for the years 2000, 2005 and 2010 where data is available for the same indicator as used for the CSDGs as well as the most recent data provided by the government line ministries and made it readily available for all at this website (https://ki.se/en/gph/research-projects) and as a Supplementary Data 1.

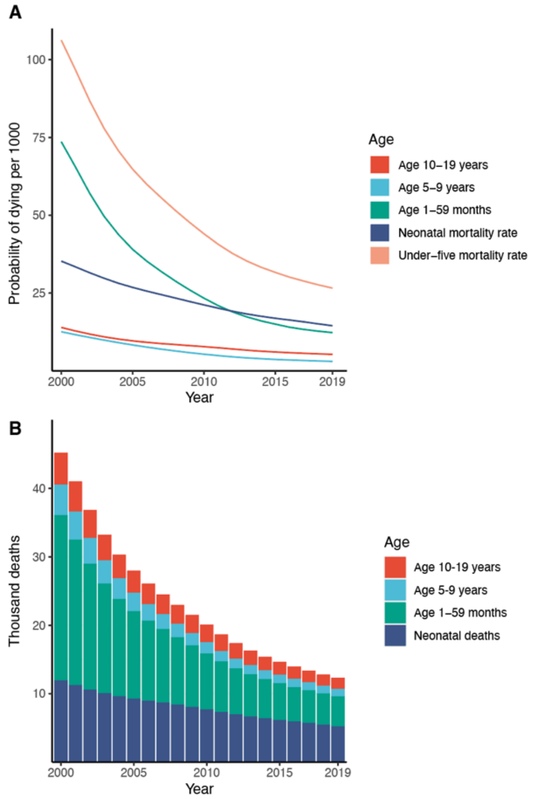

Supplementary Figure 1. Child mortality trends over time in Cambodia. 1A: Probability of dying per 1000 individuals at the start of the age. 1B: Number of deaths per age group. Data from United Nations Inter-agency Group for Child Mortality Estimation (https://childmortality.org/data/Cambodia).

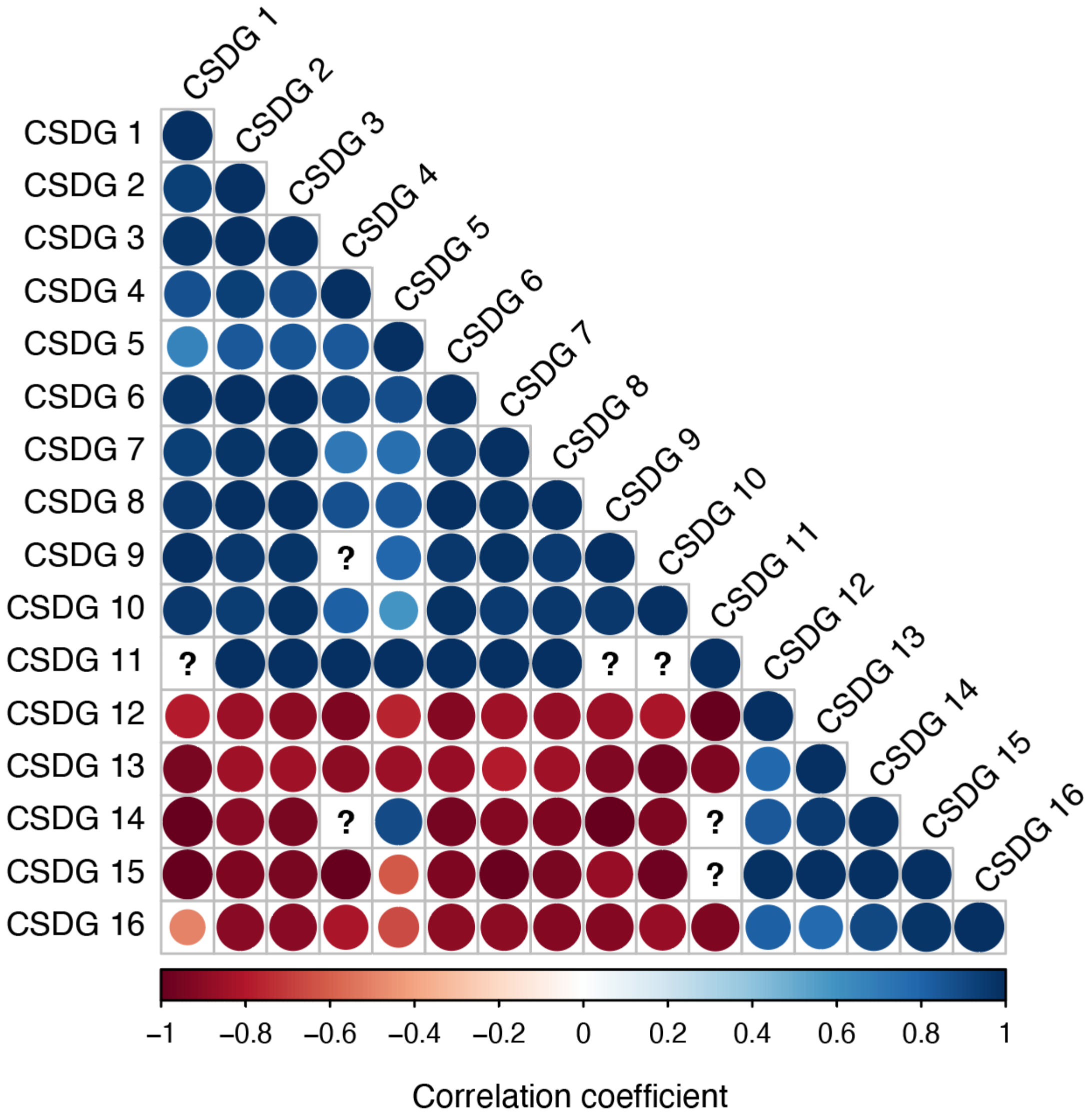
CSDG 1
CSDG 2
CSDG 3
CSDG 4
CSDG 5
CSDG 6
CSDG 7
CSDG 8
CSDG 9
CSDG 10
CSDG 11
CSDG 12
CSDG 13
CSDG 14
CSDG 15
CSDG 16
?
−1
−0.8
−0.6
−0.4
−0.2
0
0.2
0.4
0.6
0.8
1
Correlation coefficient

Supplementary Figure 2. Correlation between the Cambodian Sustainable Development Goals (CSDGs). Colour and size according to correlation coefficient scale. Question marks represents instances where no pairwise correlation could be calculated due to lack of data. Cambodia Sustainable Development Goals 1 no poverty, 2 zero hunger, 3 child health, 4 quality education, 5 gender equality, 6 clean water and sanitation, 7 affordable and clean energy, 8 decent work and economic growth, 9 industry, innovation and infrastructure, 10 reduced inequalities, 11 sustainable cities and communities, 12 responsible consumption and production, 13 climate change, 14 life below water, 15 life on land, 16 peace, justice and strong institutions.

Supplementary Figure 3. Policy overview and developmental context of Cambodia.

1993 - Set of constitution<br>1994 – National Program to Rehabilitate and Develop Cambodia<br>1996-2000 – Socioeconomic Development Plan I<br>1999 – ASEAN Free Trade Area	2001 – Land policy<br>2001 – 2005 Socio-Economic Development Plan II<br>2002 – National Poverty Reduction Strategy<br>2003 – 2015 Cambodia Millennium Development Goals<br>2004 – Cambodia part of World Trade Organization<br>2005 – 2010 Small/Medium Enterprises Development Policy<br>2006 – 2010 National Strategic Development Plan<br>2009-2015 The National Nutrition Strategy	2011 – 2015 UN Development Assistance Framework<br>2015 – Cambodia reached lower middle-income status<br>2015 – 2025 Industrial Development Plan<br>2016 – 2030 Cambodian Sustainable Development Goals<br>2018 – 2027 National multisectoral action plan for the prevention and control of noncommunicable diseases<br>2019 – 2023 National Strategic Development Plan<br>2021 – 2030 Health Strategic Plan (HSP4)

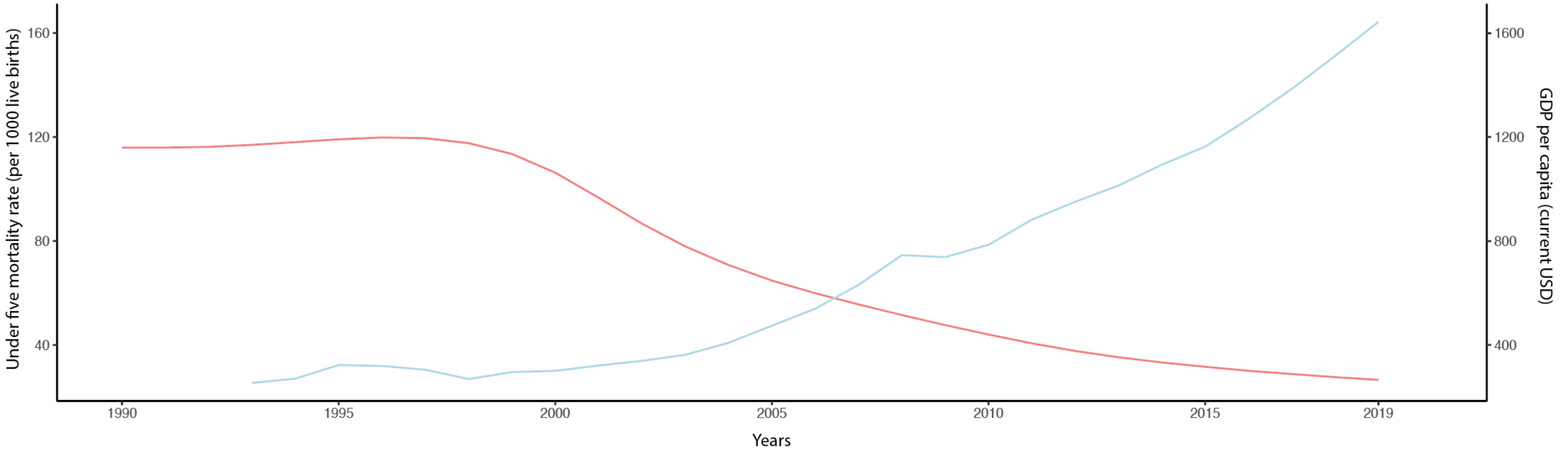

1990 – Immunization policies enacted<br>1996 -Vitamin A linked with routine immunization<br>1996 - Health Coverage Plan developed<br>1996 – Health Workforce Development Plan first developed<br>1997 – National policy on ARI/CDD and Cholera control<br>1997 – National malaria and dengue control programmes<br>1998 – Integrated Management of Childhood Illnesses (IMCI) in health centers	2002 – First national policy on child feeding<br>2003-2007 – Cambodia Nutrition Investment Plan<br>2003-2008 – Health Strategic Plan<br>2004 – Child survival benchmark review and high-level consultation<br>2005 - Sub-decree on marketing of products for infant & young child feeding<br>2006-2015 Child survival strategy<br>2008-2015 Health Strategic Plan (HSP1)	2008-2012 Strategic framework for food security and nutrition<br>2008-2015 Health Strategic Plan (HSP2)<br>2009-2015 The national nutrition strategy<br>2016-2020 Health Strategic Plan (HSP3)<br>2021 – 2030 Health Strategic Plan (HSP4)

Supplementary Note 2. Budget analysis

There is a lack of data on the government spending on health below ministry level, however ministry level data on the annual expense of line ministries has been compiled by the non-profit organization NGO forum and is available at http://www.cambodianbudget.org/index.php?page=00124.

The annual expenses from 2000-2013 in 2010 USD can be found in Supplementary Data 1, and further illustrations of the different sectors in Supplementary Figure 4 and selected ministries in Supplementary Figure 5. They showcase an increasing spending overall for social sector and the ministry of health respectively. The ministry of agriculture, forestry and fishery had a relatively downward expense trend over the years, and the ministry of women's affair saw a sharp drop in expenses between 2004 and 2005.

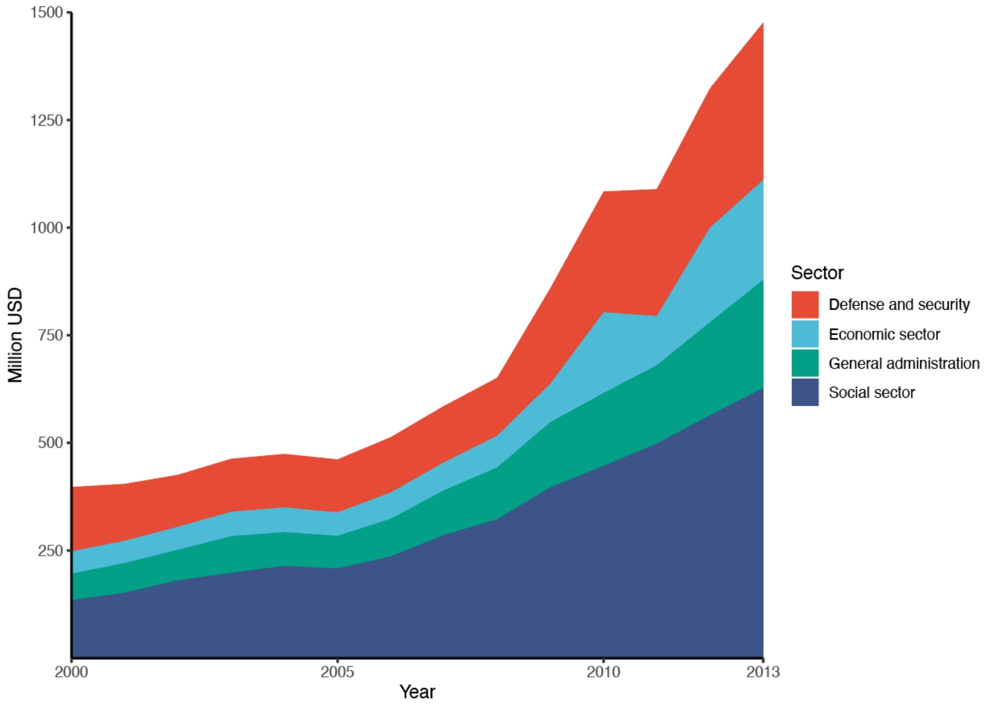

Supplementary Figure 4. Annual expenses in 2010 USD between 2000-2013.

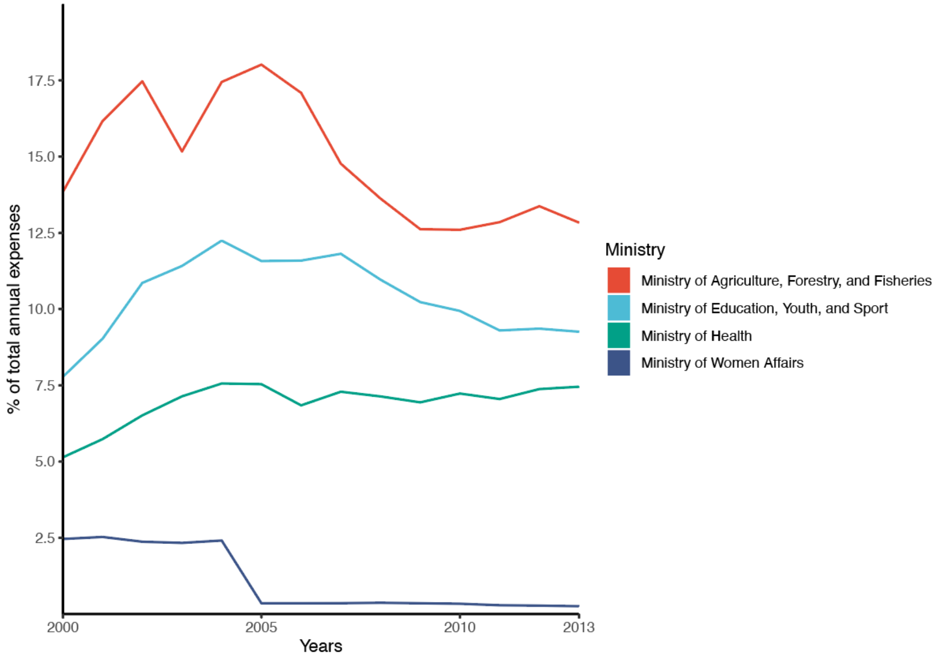

Supplementary Figure 5. Procent of total annual expenses in 2010 USD between 2000-2013 for selected ministries.

Supplementary Methods. Overivew of participants and details on the cross impact matrix and network analysis following the SDG Synergies approach.

Supplementary Table 1. List of stakeholders that participated in the study.

Name	Type of Organization	Number of participants (N=29)
National Committee for Sub-National Democratic Development	Governmental	2
Ministry of Social Affairs, Veterans and Youth Rehabilitation	Governmental	1
Ministry of Planning	Governmental	2
Ministry of Environment	Governmental	1
Cambodian Mine Action Centre	Governmental	1
Council for the Development of Cambodia	Governmental	1
Ministry of Health	Governmental	3
Ministry of Education	Governmental	1
Ministry of Women Affairs	Governmental	1
Ministry of Mines and Energy	Governmental	1
Ministry of Tourism	Governmental	1
Ministry of Labour and Vocational Training	Governmental	1
Ministry of Rural Development	Governmental	1
UNFPA	Non-governmental, global	1
Malaria Consortium	Non-governmental, global	1
Pediatric Association of Cambodia	Non-governmental, local	2
Child Right Foundation	Non-governmental, local	1
Krousar Thmey	Non-governmental, local	1
Bandos Komar	Non-governmental, local	2
Health Action Coordinating Committee	Non-governmental, local	1
Royal University of Phnom Penh UPP	Academia, local	2
Freelance consultant	Not applicable	1

Details on cross-impact matrix and network analysis

This detailed methodological section draws heavily on the work done by Weitz N, Carlsen H, Nilsson M and Skånberg K in their article "Towards systemic and contextual priority setting for implementing the 2030 Agenda" published in Sustainable Science 2018;13: 531–548.

Influence from first and second-order interactions on the network

The first simpler approach is to calculate each goal´s effect as the row sum (also called out-degree) and column sum (also called in-degree) from the cross-impact matrix (**Figure 2** in article). However, this analysis only includes direct effects, also called first order effects. If one wants to generate information that can guide prioritization of action, there is a need to account for how influence travel through the network of goals. If a goal promotes another goal which in turn has many strongly promoting interactions, its systemic impact can be very large. If the other target has few or weakly promoting interactions the positive effect however wears out quickly without having much systemic impact. Further, many strong promoting connections to other goals with the same characteristics give a high and positive multiplier effect. Conversely, a strong promoting interaction to a goal that in turn exert much negative influence on other goals makes a negative systemic impact and should be avoided. A negative interaction to a goal that in turn has strong positive connections may be a reason for caution as negative impact can spread. Due to the complexity of interpreting the ripples in the network after two interactions, we limit ourselves to consider the second-order interactions as a proxy for the systemic network effect. This can be illustrated as in Supplementary Figure 5 below.

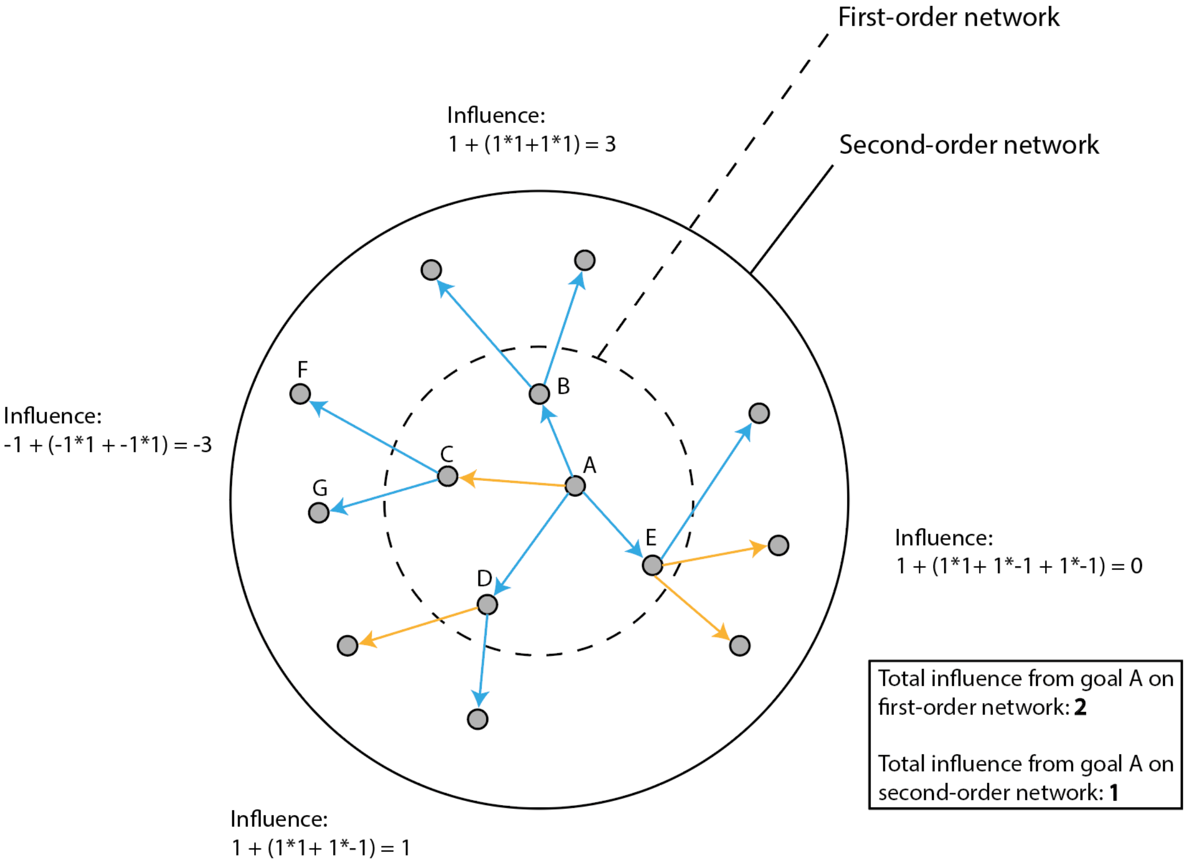

Supplementary Figure 6. Illustration of the difference between first order and second-order influence, and how second-order influence is calculated.

In the figure above, only +1 (light blue arrows) and -1 (light orange arrows) are used for simplicity. To calculate the total first-order influence of A one simply sums up the arrows in the inner circle: 3(+1) + 1(-1) = 2. To calculate the influence of A on second-order interactions we consider the full chain of influence (e.g. from A to F and G via C). Because the A to C link is negative it makes progress in C more difficult, hence the positive influence that C would exert on F and G if it would make progress is made less likely. Calculating A's influence on F we account for these effects by multiplying the link with the first order link from A to C, and thereafter adding the second-order links with the first-order links. The equation is presented below. Adding up the total influence from the four chains of influence in the figure, the total influence from target A on the second-order network is 1. Formally the net influence (I) of a goal (g) on the network as a whole including the second order interactions was calculated according to

$$1)\ I_g^{Total} = I_g^{1st} + \sum I^{2nd} = D_g^{Out} + \sum_{j \neq g} I_{gj} D_j^{Out}$$

where I_g^{1st} is the influence of goal g on its closest neighbours, I^{2nd} is the influence from g`s neighbour's on their neighbours, D_g^{Out} is the out-degree of goal g, I_{gj} is the strength of link from goal g to goal j, and D_j^{Out} is the out-degree of goal g.

Second-order interactions for individual goals

To gain a more systemic overview of the influence from individual goals on other goals, it is useful to look beyond direct (or first-order) influence and also include indirect (or second-order) influence. However, with a large number of targets included in a study, the network of interactions quickly becomes very complex. To be able to visualize second order effects, we aggregated the second order impacts stemming from progress on a particular goal. This can be illustrated in Supplementary Figure 2.

In the example, target A has a strongly promoting influence (+3) on goal B. Target B in turn has a strongly promoting influence (+3) on goal D. The indirect influence from target A on target D, mediated via target B, is thus 9. However, target A also has a weakly promoting influence (+1) on target C. Target C in turn has a weakly restricting influence (-1) on target D. The indirect influence from target A on target D, mediated via target C, is thus -1. The aggregated influence from target A on target D,

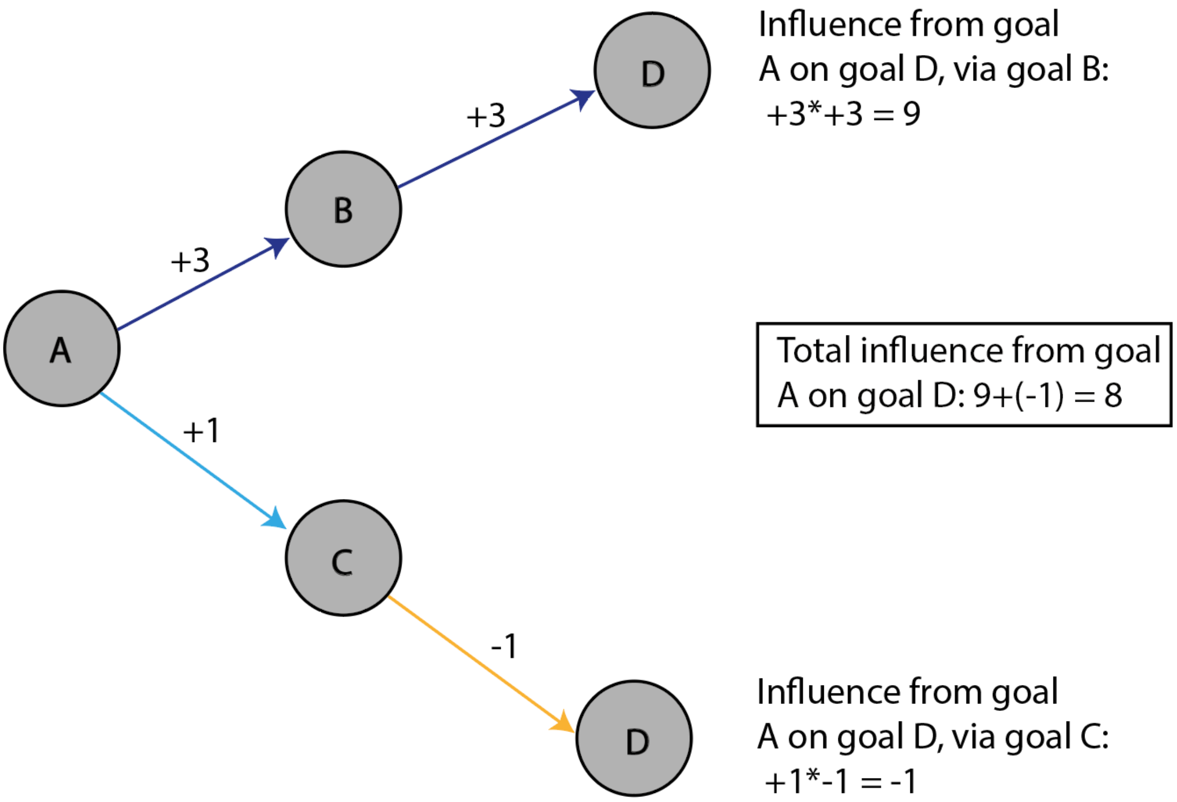

mediated via both target B and target C, is thus 8.

Supplementary Figure 7. Illustration of second-order interaction and aggregated second-order influence from goal A on goal D.

Formally, the second order influence of a goal *A* on another goal *D* is estimated by

$$2)\ I^{2nd}_{A\to D} = \sum_i w_{Ai} w_{iD}$$

where *i* runs over all goals connecting *A* and *D,* and w_{ij} is the weight on the link between goal *i* and goal
