# Supplementary material for: A stakeholder group assessment of interactions between child health and the sustainable development goals in Cambodia
Source: Commun Med (Lond). 2022 Jun 16;2:68. doi: 10.1038/s43856-022-00135-2 (PMC9203784; doi:10.1038/s43856-022-00135-2)
Supplement: Supplementary file 3 — Description of Additional Supplementary Files [file 43856_2022_135_MOESM3_ESM.pdf]

Description of Additional Supplementary Files

File name: Supplementary Data 1
Description: Cross-impact matrix data.

File name: Supplementary Data 2
Description: All available indicators for sustainable development and budget expenses for Cambodia.
