# Supplementary material for: A stakeholder group assessment of interactions between child health and the sustainable development goals in Cambodia
Source: Commun Med (Lond). 2022 Jun 16;2:68. doi: 10.1038/s43856-022-00135-2 (PMC9203784; doi:10.1038/s43856-022-00135-2)
Supplement: Supplementary file 6 — Reporting Summary [file 43856_2022_135_MOESM6_ESM.pdf]

nature research

Corresponding author(s): Daniel Helldén

Last updated by author(s): May 22, 2022

Reporting Summary

Nature Research wishes to improve the reproducibility of the work that we publish. This form provides structure for consistency and transparency in reporting. For further information on Nature Research policies, see our Editorial Policies and the Editorial Policy Checklist.

Statistics

For all statistical analyses, confirm that the following items are present in the figure legend, table legend, main text, or Methods section.

n/a	Confirmed	
☐	☒	The exact sample size (n) for each experimental group/condition, given as a discrete number and unit of measurement
☐	☒	A statement on whether measurements were taken from distinct samples or whether the same sample was measured repeatedly
☒	☐	The statistical test(s) used AND whether they are one- or two-sided<br>*Only common tests should be described solely by name; describe more complex techniques in the Methods section.*
☒	☐	A description of all covariates tested
☒	☐	A description of any assumptions or corrections, such as tests of normality and adjustment for multiple comparisons
☒	☐	A full description of the statistical parameters including central tendency (e.g. means) or other basic estimates (e.g. regression coefficient) AND variation (e.g. standard deviation) or associated estimates of uncertainty (e.g. confidence intervals)
☒	☐	For null hypothesis testing, the test statistic (e.g. F, t, r) with confidence intervals, effect sizes, degrees of freedom and P value noted<br>*Give P values as exact values whenever suitable.*
☒	☐	For Bayesian analysis, information on the choice of priors and Markov chain Monte Carlo settings
☒	☐	For hierarchical and complex designs, identification of the appropriate level for tests and full reporting of outcomes
☒	☐	Estimates of effect sizes (e.g. Cohen's d, Pearson's r), indicating how they were calculated

Our web collection on statistics for biologists contains articles on many of the points above.

Software and code

Policy information about availability of computer code

Data collection	The data collection was done using the customized publicly available digital software (https://www.sdgsynergies.org/) developed by the Stockholm Environment Institute. This is made clear in the method section.
Data analysis	The data analysis was done using the customized publicly available digital software (https://www.sdgsynergies.org/) developed by the Stockholm Environment Institute. This is made clear in the method section.

For manuscripts utilizing custom algorithms or software that are central to the research but not yet described in published literature, software must be made available to editors and reviewers. We strongly encourage code deposition in a community repository (e.g. GitHub). See the Nature Research guidelines for submitting code & software for further information.

Data

Policy information about availability of data

All manuscripts must include a data availability statement. This statement should provide the following information, where applicable:

- Accession codes, unique identifiers, or web links for publicly available datasets
- A list of figures that have associated raw data
- A description of any restrictions on data availability

Source data are included in this published article in Supplementary Data 1 and Supplementary Data 2.

Field-specific reporting

Please select the one below that is the best fit for your research. If you are not sure, read the appropriate sections before making your selection.

☐ Life sciences ☒ Behavioural & social sciences ☐ Ecological, evolutionary & environmental sciences

For a reference copy of the document with all sections, see nature.com/documents/nr-reporting-summary-flat.pdf

Behavioural & social sciences study design

All studies must disclose on these points even when the disclosure is negative.

Study description	The semi-quantitative SDG Synergies approach with a primary focus on child health was utilised in this study. This meant that the participants scored interactions between 17 Cambodian Sustainable Development Goals on a scale from -3 to +3.
Research sample	Governmental and non-governmental stakeholders in Cambodia.
Sampling strategy	The participants were purposively selected based on predefined criteria of having either expertise in child health in Cambodia, or being from a non-health sector (for example water and sanitation, agriculture, infrastructure etc.) reflecting the social, economic, political, environmental, and cultural determinants of health and working in a capacity that includes multisectoral collaboration in the country.
Data collection	All data were directly inputted into the customized publicly available digital software (https://www.sdgsynergies.org/) developed by the Stockholm Environment Institute.
Timing	The data collection took place over two days on the 24-25th of August 2020.
Data exclusions	No data were exclded.
Non-participation	No participant declined to participate.
Randomization	There were no randomization.

Reporting for specific materials, systems and methods

We require information from authors about some types of materials, experimental systems and methods used in many studies. Here, indicate whether each material, system or method listed is relevant to your study. If you are not sure if a list item applies to your research, read the appropriate section before selecting a response.

Materials & experimental systems

n/a	Involved in the study	
☒	☐	Antibodies
☒	☐	Eukaryotic cell lines
☒	☐	Palaeontology and archaeology
☒	☐	Animals and other organisms
☐	☒	Human research participants
☒	☐	Clinical data
☒	☐	Dual use research of concern

Methods

n/a	Involved in the study	
☒	☐	ChIP-seq
☒	☐	Flow cytometry
☒	☐	MRI-based neuroimaging

Human research participants

Policy information about studies involving human research participants

Population characteristics	See above (sampling strategy).
Recruitment	The participants were purposively selected based on predefined criteria of having either expertise in child health in Cambodia, or being from a non-health sector (for example water and sanitation, agriculture, infrastructure etc.) reflecting the social, economic, political, environmental, and cultural determinants of health and working in a capacity that includes multisectoral collaboration in the country. This might have led the participants to score more favorably for their own sector specific goals.
Ethics oversight	The study received ethical approval from the National Ethics Committee for Health Research Cambodia (NECHR-023) and written informed consent was obtained from all participants.

Note that full information on the approval of the study protocol must also be provided in the manuscript.
